# *Roseomonas* sp. Isolated from Ticks, China

**DOI:** 10.3201/eid1607.090166

**Published:** 2010-07

**Authors:** Wei Liu, Fang Zhang, Er-Chen Qiu, Jun Yang, Zhong-Tao Xin, Xiao-Ming Wu, Fang Tang, Hong Yang, Wu-Chun Cao

**Affiliations:** Author affiliations: Beijing Institute of Microbiology and Epidemiology, Beijing, People’s Republic of China (W. Liu, F. Zhang, E.-C. Qiu, X.-M. Wu, H. Yang, W.-C. Cao);; Chinese People’s Armed Police Force Center for Disease Control and Prevention, Beijing (J. Yang, F. Tang);; Chinese National Human Genome Center, Beijing (Z.-T. Xin)

**Keywords:** Roseomonas, bacteria, ticks, Dermacentor nuttalli, China, letter

**To the Editor:** *Roseomonas*, which produces pink colonies, is a newly described genus of gram-negative bacteria (*1*). Human infections with *Roseomonas* spp. have been reported in the past decade, mostly in immunocompromised persons with underlying diseases such as acute leukemia, cancer, and rheumatoid arthritis (*2**–**5*). A healthy woman was reported to be infected by *R. gilardii* after being bitten by a spider (*6*), which indicated possible transmission by an arthropod.

As a part of an investigation of tick-borne diseases, we collected actively questing and feeding ticks in Xinjiang Autonomous Region, People’s Republic of China, in the summers of 2007 and 2008 (*7*). Ticks were washed in 75% ethanol, 30% hydrogen peroxide, and sterile distilled water. Five ticks of the same species, sex, and developmental stage were pooled and ground in 1 mL of saline. A 0.1-mL suspension was placed on cysteine heart agar plates containing chocolate and 9% sheep blood (Becton Dickinson Microbiology Systems, Cockeysville, MD, USA) and supplemented with colistin, amphotericin, lincomycin, trimethoprim, and ampicillin. Eggs laid by engorged female ticks were collected and kept at room temperature.

Fourteen days after hatching, larval ticks were processed as a batch by using the same methods described above. After 2–3 days of incubation at 37°C, pink colonies were observed in 9 cultures, 8 of which originated from engorged female *Dermacentor nuttalli* ticks. The other culture originated from larval ticks, the progeny of an engorged female *D. nuttalli* tick. Colonies were pinpoint, pale pink, shiny, raised, and mucoid. The pink color of the colonies became pronounced when the bacteria were transferred onto plates containing Luria-Bertani agar. Bacteria were gram-negative, plump, coccoid rods, in pairs or short chains. Electron microscopy showed that each organism was ≈0.7 × 1.1 μm.

The 9 isolates showed identical phenotypic and biochemical characteristics, which were similar to those of previously reported *Roseomonas* spp (*1*). However, the isolates required a lower salt concentration (<4% NaCl) and a higher temperature (37°C instead of <35°C) for optimal growth than other *Roseomonas* spp. Antimicrobial drug susceptibility tests showed that the isolates were susceptible to aminoglycosides (amikacin, gentamicin, and tobramycin), tetracycline, and a β-lactam (imipenem) and resistant to cephalosporins (similar to *R*. *cervicalis*) (*1*) and sulfamethoxazole.

To further characterize the *Roseomonas* sp. isolated in this study, we amplified and sequenced the 16S rRNA gene. Sequences of the 9 isolates were identical to each other and showed 98%–99.1% similarity with reported species within the genus *Roseomonas*. A phylogenetic tree based on 16S r RNA genes (Figure) showed that the *Roseomonas* sp. identified in this study (representative strain XTD 510, GenBank accession no. EU742165) was in the same branch as *R. cervicalis* ATCC 49957 (GenBank accession no. AY150047). The new isolate was not genetically related to *R*. *fauriae* and *R*. *genespecies* 6, which have been reported as not belonging to the genus *Roseomonas* (*8*). The new isolate was also distinct from 2 other species from China, *R*. *lacus* TH-G33 (GenBank accession no. AJ 78600), which was isolated from freshwater lake sediment (*9*), and *Roseomonas* sp. JS018 (GenBank accession no. DQ 010108), which was isolated from soil (*10*).

**Figure:**
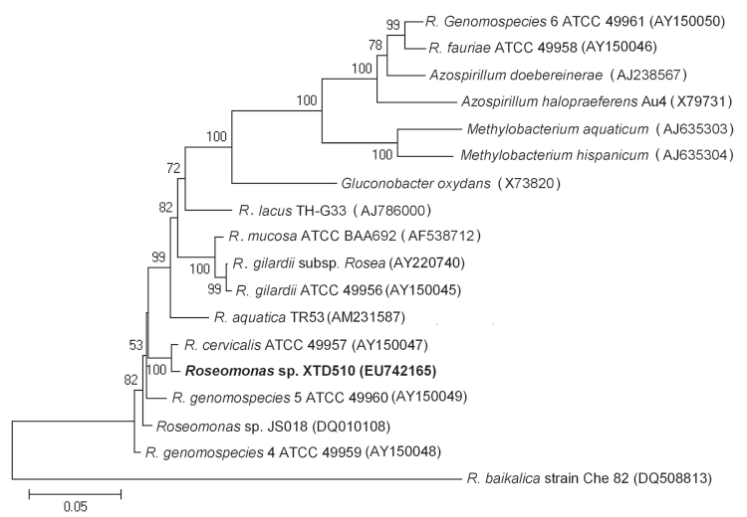
Unrooted phylogenetic tree based on 16S rRNA gene sequences of *Roseomonas* spp. Tree was constructed by using MEGA 4.0 software (www.megasoftware.net) and the neighbor-joining method with 1,000 bootstrap replicates. Genetic distances were calculated by using the Kimura 2-parameter correction at the nucleotide level. Bootstrap values >50% are shown. The isolate obtained in this study is shown in **boldface**. GenBank accession numbers of reference strains are marked after each strain name. Scale bar indicates nucleotide substitutions per site.

We isolated a novel *Roseomonas* sp. from adult *D. nuttalli* ticks and their larval progeny and obtained evidence of transovarial transmission. Although we cannot conclude that ticks are vectors or reservoirs of *Roseomonas* spp., their roles in transmitting the bacteria deserve further study. *D. nuttalli* ticks are a dominant species in the study area and usually parasitize a variety of wild and domestic animals. These ticks often feed on humans as alternative hosts. Because this *Roseomonas* sp. is not a common pathogen, its role in public health and veterinary medicine is unkown.

Phenotypic characterization of the isolates indicated similarities with previously reported *Roseomonas* spp. Phylogenetic analysis showed that the novel *Roseomonas* sp. is closely related to *R*. *cervicalis*, which was isolated from a cancer patient. Our isolates also differed from 2 reported strains isolated from freshwater lake sediment in Jiangsu Province, China (*9*) and from soil in Fujian Province, China (*10*). This result indicated the species diversity of the genus *Roseomonas*, which might be related to different bacterial origins. Because of the unique biochemical characteristics, antimicrobial drug susceptibilities, and novel isolation source of our isolates, the pathogenesis of this organism should be investigated.
